# Effects of respiratory muscle training on respiratory function, functional outcomes, and pulmonary complications in patients with stroke: a systematic review and meta-analysis of randomized controlled trials

**DOI:** 10.3389/fneur.2026.1981476

**Published:** 2026-10-23

**Authors:** Ying Guo, Yanping Shang, Li Tian, Wen Li, Siwen Zhu, Jing Jia

**Affiliations:** Department of Cardiopulmonary Rehabilitation, Guangdong Provincial Work Injury Rehabilitation Hospital, Guangzhou, China

**Keywords:** meta-analysis, pneumonia, randomized controlled trial, respiratory function, respiratory muscle training, subacute stroke

## Abstract

**Objective:**

To systematically evaluate the effects of respiratory muscle training on respiratory function, functional outcomes, and pulmonary complications in patients with subacute stroke.

**Method:**

We systematically searched PubMed, EMBASE, Cochrane Library, Web of Science, Scopus, and ClinicalTrials.gov up to August 12, 2026 for randomized controlled trials (RCTs). Pooled estimates were calculated using Hartung-Knapp-adjusted random-effects models. Two reviewers independently screened, extracted data, and assessed risk of bias (ROB2). Sensitivity and publication bias were evaluated via leave-one-out and trim-and-fill analyses.

**Results:**

Eleven RCTs (Final analysis sample:737 patients) were included. Respiratory muscle training significantly improved six-minute walk distance (mean difference [MD] = 34.09 m, 95% confidence interval [CI] 9.71–58.47), forced expiratory volume in one second (MD = 0.32 L, 95% CI 0.16–0.49), forced vital capacity (MD = 0.31 L, 95% CI 0.20–0.41), maximum inspiratory pressure (MD = 14.16 cmH₂O, 95% CI 6.93–21.38), and Trunk Impairment Scale scores (MD = 2.27, 95% CI 1.12–3.43). Respiratory muscle training also reduced pneumonia incidence (risk ratio = 0.41, 95% CI 0.24–0.72). Heterogeneity was substantial for maximum inspiratory pressure (I^2^ = 89.9%), moderate-to-high for forced expiratory volume in one second (I^2^ = 62.4%), and moderate for Trunk Impairment Scale scores (I^2^ = 56.0%); forced vital capacity showed no heterogeneity (I^2^ = 0%). Sensitivity analyses indicated that the six-minute walk test and Trunk Impairment Scale findings were sensitive to individual trials, whereas forced expiratory volume in one second, forced vital capacity, and maximum inspiratory pressure estimates remained statistically significant after leave-one-out analyses. Egger’s test for maximum inspiratory pressure was not significant (*p* = 0.097), but trim-and-fill analysis suggested potential missing studies for forced expiratory volume in one second, forced vital capacity, and maximum inspiratory pressure.

**Conclusion:**

In patients with subacute stroke, respiratory muscle training is associated with improvements in respiratory function and functional outcomes and with a reduced risk of pneumonia. However, some pooled estimates were sensitive to individual trials and the evidence remains limited by heterogeneity, indicating a need for further large randomized trials.

**Systematic review registration:**

https://www.crd.york.ac.uk/PROSPERO/view/CRD420261482562, Identifier, CRD420261482562.

## Introduction

Stroke remains a leading cause of mortality and long-term disability worldwide, with approximately 12.2 million incident cases and 101 million prevalent cases reported in 2019 (1, 2). Although advances in acute stroke management have reduced case-fatality rates (3), the majority of survivors are left with persistent functional impairments, with only 50–70% achieving good functional recovery and 15–30% remaining permanently disabled at 3 months post-onset (4, 5). This substantial residual disability underscores the critical need for effective rehabilitation interventions.

Among the myriad complications following stroke, respiratory dysfunction is a prevalent yet frequently underrecognized consequence. Stroke-induced neurological damage impairs the neural control and contractile function of respiratory muscles, including the diaphragm, intercostal muscles, and abdominal muscles (6, 7). The pathophysiological mechanisms encompass direct injury to corticospinal pathways projecting to respiratory motor neurons, reduced central respiratory drive, diaphragmatic atrophy, and chest wall stiffness from hemiplegic posturing (8–10). The clinical consequences are profound: stroke patients exhibit significant respiratory muscle weakness, with mean maximal inspiratory and expiratory pressures being approximately 25–33% lower than those of healthy controls (11); moreover, up to 89% of patients show inspiratory and 82.6% show expiratory muscle weakness when defined as pressures below 70% of predicted values (12). These deficits translate into reduced lung volumes (FVC and FEV₁), diminished cough effectiveness, and impaired airway clearance (13, 14).

The clinical significance of post-stroke respiratory dysfunction extends beyond pulmonary physiology. Diminished respiratory muscle strength is independently associated with reduced exercise tolerance, impaired trunk control and balance, increased fatigue during daily activities, and heightened risk of pulmonary complications. In stroke survivors, lower respiratory muscle strength and pulmonary function correlate with worse trunk control and balance and respond to respiratory muscle training with cross-domain improvements (15, 16). In both stroke and other chronic conditions such as heart failure and COPD, inspiratory muscle weakness is linked to limited exercise capacity and heightened dyspnoea, which in turn reduces daily activity and contributes to fatigue (17–19). Moreover, decreased maximal inspiratory pressure and impaired cough strength are associated with increased risk of pneumonia and respiratory failure (20, 21). Critically, stroke-associated pneumonia (SAP) is a serious complication, affecting 7–12% of patients, and is independently associated with markedly increased mortality (up to ~6-fold during hospitalization and ~2- to 3-fold at 30 days and 1 year) as well as prolonged hospital stay (22, 23). Interventions that can reduce pneumonia incidence represent a high-priority clinical objective.

Respiratory muscle training (RMT) has emerged as a promising rehabilitative intervention. RMT encompasses structured breathing exercises—inspiratory muscle training (IMT), expiratory muscle training (EMT), or their combination—performed using hand-held devices providing pressure-threshold or flow-dependent resistance (24). The physiological rationale is grounded in the principle that respiratory muscles, like other skeletal muscles, respond to overload through structural and functional adaptations, including increased fiber cross-sectional area, enhanced mitochondrial density, and improved neural drive (25).

Over the past decade, accumulating RCTs have examined RMT effects in stroke populations, yet prior meta-analyses have yielded inconsistent conclusions. Fabero-Garrido et al. (26) reported improved exercise tolerance and pulmonary function but no significant effect on FEV₁, with benefits not sustained at medium-term follow-up. Menezes et al. (27) provided early evidence of respiratory complications reduction, but a subsequent corrigendum (28) revised their pneumonia estimate from significant to non-significant. Wu et al. (29) reported respiratory complications reduction (OR 0.55, *p* = 0.050), leaving the evidence inconclusive. The Cochrane review by Xiao et al. (30), based on only two small trials (*n* = 66), concluded insufficient evidence for IMT—a conclusion now outdated. Gomes-Neto et al. (31) and Pozuelo-Carrascosa et al. (32) reported positive effects on respiratory function and walking capacity but did not separately examine pneumonia or trunk control. Liu et al. (33) confirmed MIP and 6MWT improvements but reported high heterogeneity (I^2^ = 92%), and Li et al. (17) found threshold RMT improved MIP and MEP but not PEF. Huang et al. (34) specifically examined chronic stroke and found RMT improved MIP but failed to improve MEP, FVC, or exercise capacity, suggesting efficacy attenuates with time since stroke.

Collectively, prior meta-analyses have critical limitations: (1) they predominantly pooled patients across all stroke phases, obscuring phase-specific effects; (2) they did not systematically differentiate volume-based (FVC) from pressure-based (MIP) outcomes; (3) they provided weak or inconclusive evidence for pneumonia prevention; and (4) they did not examine trunk control as a functional outcome. The subacute stroke phase (2 weeks to 6 months) represents a critical neuroplastic window during which rehabilitation interventions likely yield maximal benefit (35, 36), yet no meta-analysis has specifically focused on this population.

Therefore, this meta-analysis was undertaken to: (1) systematically evaluate RMT effects on respiratory function, functional outcomes, and pneumonia incidence specifically in subacute stroke patients; (2) examine the differential heterogeneity between FVC and MIP to clarify the consistency of RMT-induced adaptations; (3) provide the first robust quantitative synthesis of pneumonia prevention; and (4) quantitatively synthesize Trunk Impairment Scale outcomes, linking respiratory training to trunk control for the first time. We hypothesized that RMT would significantly improve all examined outcomes, with FVC demonstrating lower heterogeneity than MIP, and that pneumonia incidence would be significantly reduced, establishing RMT as a clinically meaningful component of subacute stroke rehabilitation.

## Methods

### Study design

This meta-analysis adhered to the 2020 PRISMA guidelines (37) and was registered with PROSPERO (CRD420261482562) after completion of the initial literature search but before all subsequent review stages. Specifically, the literature search across all databases was completed on August 12, 2026. The study protocol was subsequently submitted to PROSPERO on August 19, 2026, and was reviewed and formally registered on the same day. It should be noted that only the initial search had been completed at the time of PROSPERO submission; title/abstract screening, full-text eligibility assessment, risk of bias assessment, and data extraction were all conducted strictly in accordance with the protocol after formal registration.

### Search strategy

PubMed, Embase, Web of Science, Scopus, and the Cochrane Library were searched from inception to 12 August 2026, supplemented by ClinicalTrials.gov for ongoing or completed trials. Core search terms combined “stroke,” and “respiratory muscle training”. Detailed strategies are provided in the Supplementary materials.

### Inclusion and exclusion criteria

Inclusion criteria were as follows: (1) Participants: Adult patients aged 18 years or older with a diagnosis of stroke (ischemic or hemorrhagic) who were in the subacute phase, defined as 7 days to 6 months after stroke onset, or as defined by the trial investigators. (2) Intervention: Respiratory muscle training (inspiratory, expiratory, or combined), delivered alone or as an adjunct to usual rehabilitation. Any training modality was eligible (e.g., threshold loading, resistive loading, normocapnic hyperpnea), with no restrictions on intensity, frequency, session duration, or treatment duration. (3) Comparator: Control group receiving no respiratory muscle training, including usual care, conventional rehabilitation, sham training, or no intervention. (4) Outcomes: Reporting at least one of the following outcomes: respiratory function parameters (e.g., forced expiratory volume in one second, forced vital capacity, maximum inspiratory pressure, maximum expiratory pressure), functional outcomes (e.g., six-minute walk distance, Trunk Impairment Scale score), or pulmonary complications (e.g., pneumonia incidence). Pneumonia, broadly defined (pneumonia or lung infection), i.e., pneumonia or lung infection as reported in the original studies, together with respiratory complications for which these two conditions constitute the core components. (5) Study design: Randomized controlled trials.

The exclusion criteria are as follows: (1) Other types of articles, such as case reports, case–control study, conference, publications, thesis, letters, comments, reviews, meta-analyses, editorials, protocols, retrospective study, etc. (2) Acute or chronic stroke. (3) Not relevant. (4) Duplicate publications. (5) The datas were not reported.

### Selection of articles

After removing duplicates in EndNote (Version 20), two reviewers independently screened titles/abstracts and assessed full texts against eligibility criteria. When outcome data were missing from primary articles, Supplementary materials were consulted. Disagreements were resolved by consensus, with arbitration by a third author if necessary.

### Data extraction

Two reviewers independently extracted data using a standardized form: study characteristics (region, year, first author, trial number), baseline demographics and clinical features (sample size, age, type of stroke, time-window), and outcomes. Discrepancies were settled through discussion with a third investigator.

### Quality assessment

Risk of bias was evaluated using the Cochrane Risk of Bias 2.0 (ROB2) (38) tool, covering the randomization process, deviations from intended interventions, missing outcome data, measurement of the outcome, and selection of the reported result. Each domain was rated as low risk, some concerns, or high risk. Any discrepancies were resolved by consensus.

### GRADE evidence assessment

Evidence certainty was rated using GRADE; all outcomes were downgraded one level for imprecision due to limited trial numbers.

### Statistical analysis

All meta-analyses were performed in R version 4.6.0 (RStudio 2026.04.0 + 526) using the meta, metasens, and grid packages. For continuous outcomes, treatment effects were expressed as mean differences (MDs) with 95% confidence intervals (CIs); for dichotomous outcomes, risk ratios (RRs) with 95% CIs were calculated. Given the limited number of included randomized controlled trials (*n* = 11), primary pooled estimates were calculated using the Hartung-Knapp-corrected DerSimonian-Laird random-effects model irrespective of the observed heterogeneity. As complementary analyses, inverse variance fixed-effect models were used for continuous outcomes and Mantel–Haenszel fixed-effect models for dichotomous outcomes. For dichotomous outcomes with zero events in one arm, a continuity correction of 0.5 was applied.

Between-study heterogeneity was quantified using Cochran’s Q test and the I^2^ statistic, with I^2^ values of approximately 25%, 50%, and 75% interpreted as low, moderate, and high heterogeneity, respectively. Leave-one-out sensitivity analyses were conducted by iteratively omitting one study at a time to evaluate the influence of individual trials on the pooled estimates. Publication bias was assessed using the trim-and-fill method because the number of included studies was insufficient for Egger’s test. A two-sided *p* value < 0.05 was considered statistically significant.

## Results

### Search results

A total of 1,649 studies were initially identified. After removing 471 duplicates primarily via the automatic deduplication function of EndNote software, 1,178 studies remained. Ineligible study types (e.g., review articles, protocols, case reports, animal studies, meta-analyses, and case–control studies) were excluded, leading to the removal of 889 irrelevant publications. Following manual title and abstract screening, an additional 254 articles were excluded. Finally, after full-text review, and Supplementary material retrieval, 11 randomized controlled trials met the eligibility criteria (Figure 1).

**Figure 1:**
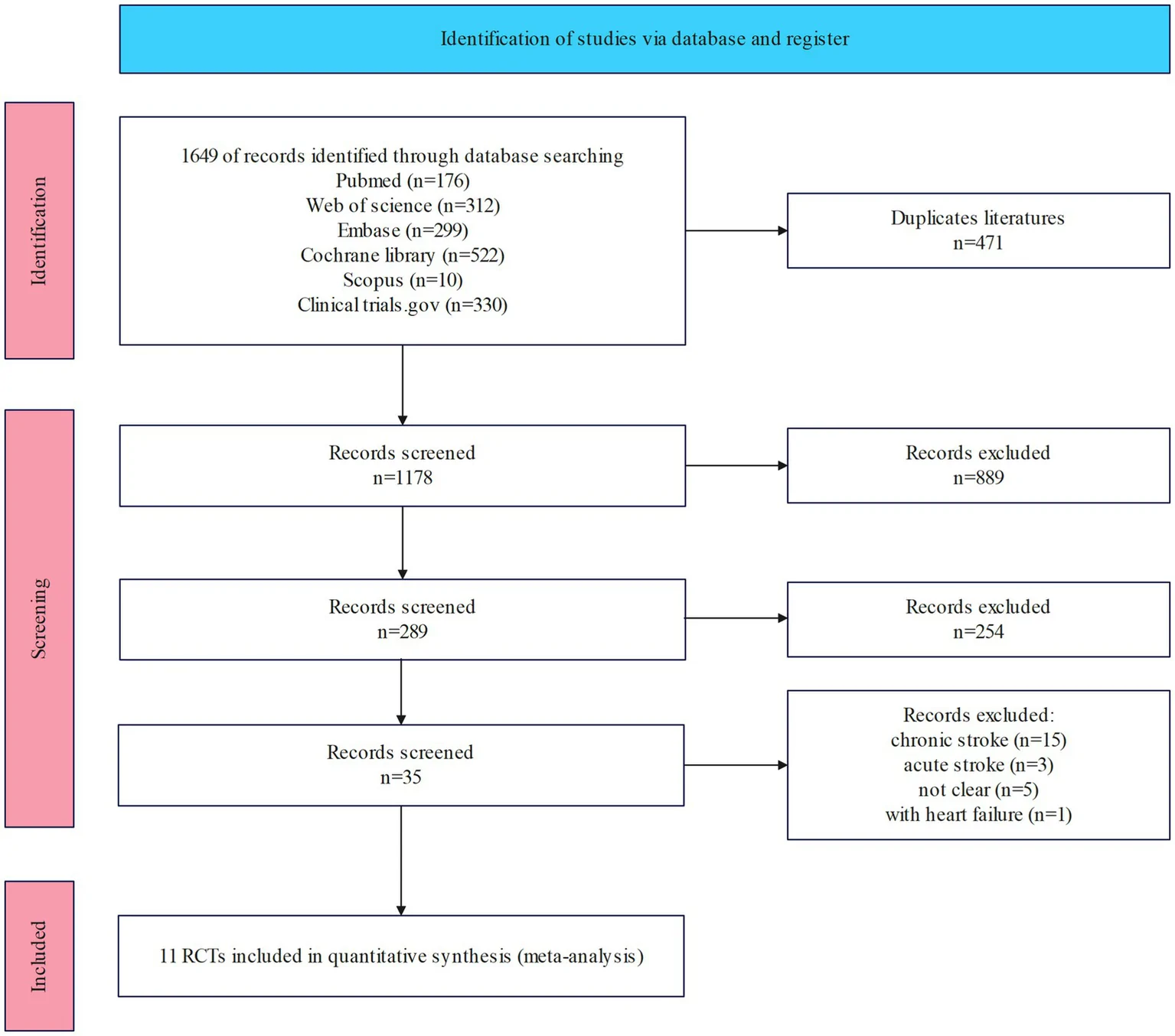
PRISMA flow diagram.

### Patient characteristics

A total of 11 randomized controlled trials involving 737 participants (Final analysis sample) with subacute stroke were included in this meta-analysis. The mean age of participants ranged from 51 to 69 years across the included trials. All participants were enrolled within 6 months of stroke onset, consistent with the subacute phase of stroke recovery. Stroke type and baseline functional status were variably reported across studies; therefore, a pooled description of these characteristics was not feasible. Overall, the included population represented adult patients in the subacute stage of stroke undergoing rehabilitation. Comprehensive baseline characteristics are presented in Table 1.

**Table 1:** Baseline characteristics of studies and participants included.

Author, year	Country	Trial number	Total enrolled sample	Group (I/C)	Age (mean/ range)	Time-window classification, d(days)	Stroke type (Ischemic %)	Lesion side (Right %)
Messaggi-Sartor (2015) (12)	Spain	NCT02125760	109	56/53	I:65.6 ± 11.4 C:67.6 ± 10.9	Early Subacute (≤30d)	100	56
Guillén-Solà (2016) (61)	Spain	NCT02473432	62	20/21/21	I:67.9 ± 10.6 70.3 ± 8.4 C:68.9 ± 7.0	Early Subacute (≤30d)	100	41.9
Sutbeyaz (2010) (62)	Turkey	NA	45	15/15/15	I:60.8 ± 6.8 62.8 ± 7.2 C:61.9 ± 6.15	Late Subacute (≥90d)	73.33	73.33
Pyun (2017) (63)	Korea	NCT03153345	40	20/20	I:57 (34–82) C:65 (34–86)	Early Subacute (≤30d)	57.5	50
Bang (2017) (64)	Korea	NA	12	6/6	I:61.2 ± 4.2 C:62.2 ± 5.3	Late Subacute (>60d)	NA	58.33
Zhu (2024) (65)	China	ChiCTR2100051492	50	25/25	I:64.36 ± 13.64 C:61.28 ± 14.95	Late Subacute (>60d)	82	40
Defi (2021) (66)	Indonesia	NA	32	16/16	I:51.37 ± 6.009 C:53.50 ± 3.864	Late Subacute (>60d)	25	6.25
Liu (2026) (67)	China	NCT06640101	88	30/28/30	I:58.0 ± 9.0 58.0 ± 11.0 C:60.0 ± 12.0	Late Subacute (>60d)	65.91	38.64
Akçay (2026) (68)	Turkey	NCT06210516	22	11/11	I: 63.27 ± 8.24 C: 63 ± 12.38	Late Subacute (>60d)	100	31.81
Zhao (2024) (69)	China	NA	329	164/165	I:61.3 ± 8.8 C:60.6 ± 8.6	Early Subacute (≤30d)	44.68	42.25
Tovar-Alcaraz (2021) (70)	Spain	NCT03899485	16	8/8	I:58.0 ± 12.9 C:56.0 ± 9.2	Late Subacute (>60d)	68.75	56.25

I: intervention group, accept respiratory muscle training. C: control group, traditional rehabilitation or others.

### Risk of bias and evidence certainty assessment

Methodological quality of the included trials was assessed using the Cochrane Risk-of-Bias tool, and a summary is shown in Figure 2. The GRADEpro GDT framework was applied to assess the certainty of evidence for selected outcomes. Complete GRADE evidence profiles are provided in the Supplementary materials.

**Figure 2:**
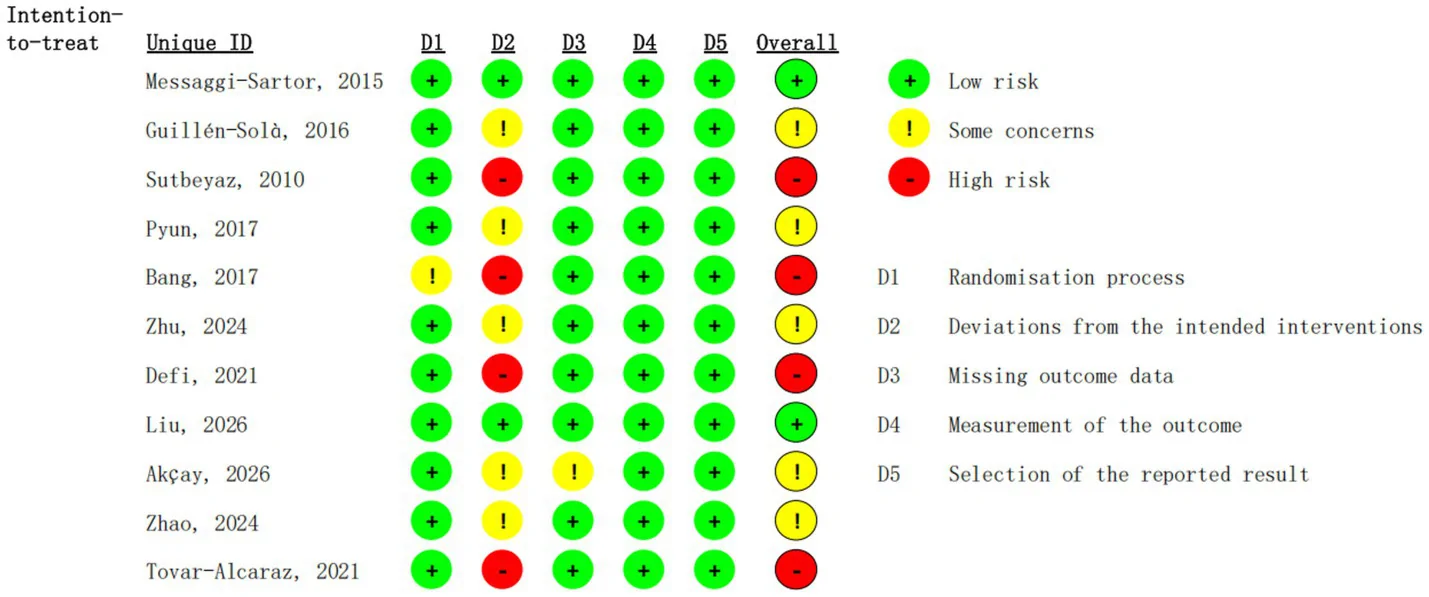
Risk of bias analysis of the included RCTs using Cochrane Rob 2.

### Maximum inspiratory pressure

Ten randomized controlled trials involving 715 participants (356 intervention, 359 control) provided data on maximum inspiratory pressure (MIP). Using the Hartung-Knapp corrected random-effects model, we found that RMT significantly improved MIP compared with control (MD = 14.16 cmH₂O, 95% CI 6.93 to 21.38, *p* < 0.001, Table 2; Figure 3). Substantial between-study heterogeneity was observed (I^2^ = 89.9%, tau^2^ = 103.29, Q-test *p* < 0.0001). Leave-one-out sensitivity analyses confirmed that the pooled estimate remained statistically significant and directionally consistent after excluding each trial individually; however, heterogeneity persisted across all iterations, indicating that the variation was driven by multiple studies rather than by a single outlier. Publication bias assessment yielded a non-significant Egger’s test (*p* = 0.097), while the trim-and-fill method suggested three potentially missing unpublished studies.

**Table 2:** Summary of the forest plot results.

Outcome	k	Total patients (E/C)	Fixed-effect model MD/RR (95% CI), *p*-value	Random-effects model (DL + HK) MD/RR (95% CI), *p*-value	Heterogeneity I^2^ (p)	τ^2^
Maximum inspiratory pressure	10	715 (356/359)	7.964 (6.341, 9.587), < 0.0001	14.156 (6.930, 21.382), 0.0001	89.9% (0.0001)	103.29
Forced expiratory volume in one second	6	226 (112/114)	0.269 (0.185, 0.352), < 0.0001	0.322 (0.156, 0.488), 0.0001	62.4% (0.0208)	0.0234
Forced vital capacity	6	226 (112/114)	0.306 (0.200, 0.411), < 0.0001	0.306 (0.200, 0.411), < 0.0001	0% (0.4343)	0
Six-minute walk test	4	152 (75/77)	34.165 (17.589, 50.741), < 0.0001	34.092 (9.714, 58.471), 0.0061	36.1% (0.1956)	210.42
Pneumonia incidence	4	511 (258/253)	0.402 (0.234, 0.693), 0.001	0.414 (0.239, 0.716), 0.0016	0% (0.7641)	0
Trunk impairment scale	3	447 (222/225)	2.320 (1.576, 3.064), < 0.0001	2.274 (1.122, 3.426), 0.0001	56.0% (0.1032)	0.58

I: intervention group, accept respiratory muscle training. C: control group, traditional rehabilitation or others.

**Figure 3:**
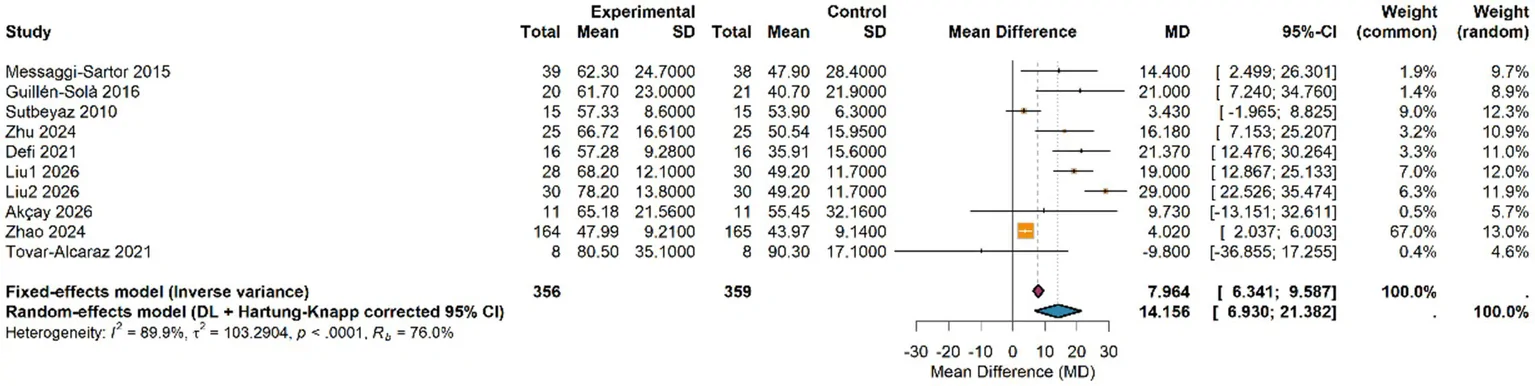
Forest plot of maximum inspiratory pressure.

### Forced expiratory volume in one second

Six RCTs involving 226 participants (112 intervention, 114 control) reported forced expiratory volume in one second (FEV₁). Using the Hartung-Knapp corrected random-effects model, we found that RMT significantly improved FEV₁ compared with control (MD = 0.322 L, 95% CI 0.156 to 0.488, *p* < 0.001, Table 2; Figure 4). Moderate-to-high between-study heterogeneity was observed (I^2^ = 62.4%, tau^2^ = 0.0234, Q-test *p* = 0.0208). Leave-one-out sensitivity analyses confirmed that the pooled estimate remained statistically significant and directionally consistent after excluding each trial individually, indicating robustness. Publication bias assessment was limited as Egger’s test was not applicable due to the small number of studies (<10); however, trim-and-fill analysis suggested one potentially missing unpublished study.

**Figure 4:**
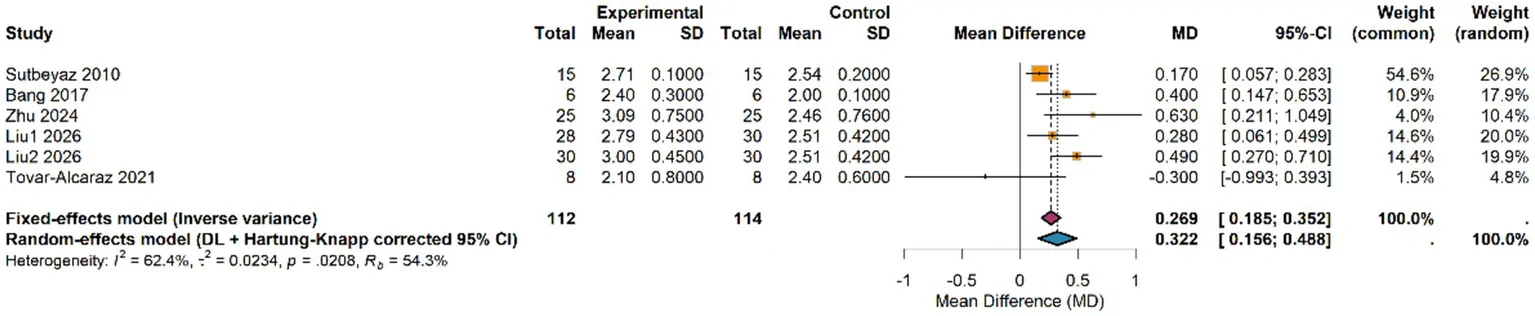
Forest plot of forced expiratory volume in one second.

### Forced vital capacity

Six RCTs with 226 participants (112 intervention, 114 control) provided data on forced vital capacity (FVC). The Hartung-Knapp corrected random-effects model yielded a significant pooled MD of 0.306 L (95% CI 0.200 to 0.411, *p* < 0.0001, Table 2; Figure 5). No between-study heterogeneity was detected (I^2^ = 0%, tau^2^ = 0, Q-test *p* = 0.4343). Leave-one-out sensitivity analysis showed that the effect remained significant after omitting any single trial, confirming high robustness. As with FEV₁, Egger’s test was not performed due to the limited number of studies, while trim-and-fill analysis suggested one potentially missing unpublished study.

**Figure 5:**
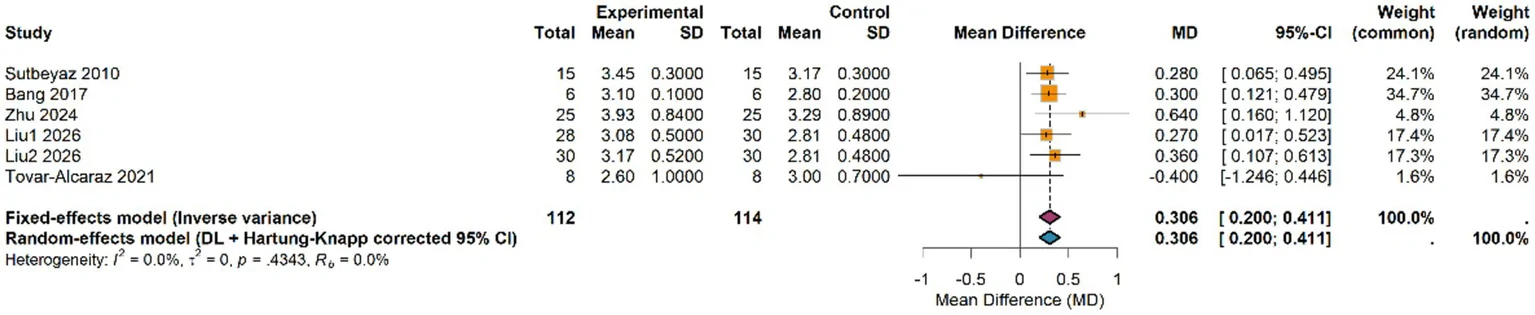
Forest plot of forced vital capacity.

### Six-minute walk test

Four randomized trials involving 152 participants (75 intervention, 77 control) reported six-minute walk test (6MWT) data. Respiratory muscle training was associated with a statistically significant improvement in walking distance (pooled MD = 34.092 m, 95% CI 9.714–58.471, *p* = 0.0061, Table 2; Figure 6). Heterogeneity was low-to-moderate (I^2^ = 36.1%; Q-test *p* = 0.1956; tau^2^ = 210.42). In leave-one-out sensitivity analysis, the pooled effect lost statistical significance after omitting Liu (MD = 19.493 m, 95% CI − 3.183 to 42.170, *p* = 0.0920), indicating that the overall finding was sensitive to this single study. Publication bias was not assessable given the limited number of included studies.

**Figure 6:**
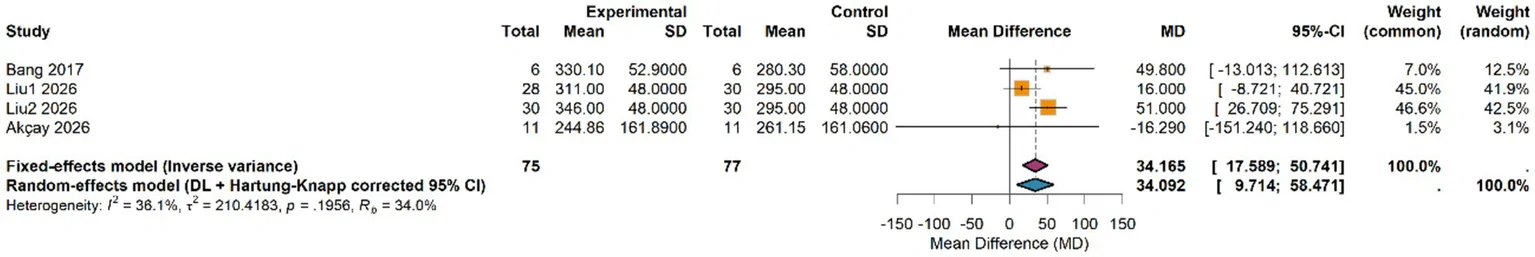
Forest plot of six-minute walk test.

### Pneumonia incidence

Four RCTs (*n* = 511; total events = 56) reported pneumonia incidence. A continuity correction of 0.5 was applied for a trial with a zero-event cell. Respiratory muscle training significantly reduced the risk of pneumonia (pooled RR = 0.414, 95% CI 0.239–0.716, *p* = 0.0016, Table 2; Figure 7). No between-study heterogeneity was detected (I^2^ = 0.0%; Q-test *p* = 0.7641). Leave-one-out sensitivity analysis confirmed the stability of this finding, with the pooled effect remaining statistically significant in all analyses. Publication bias was not reliably assessed because only four trials were available.

**Figure 7:**
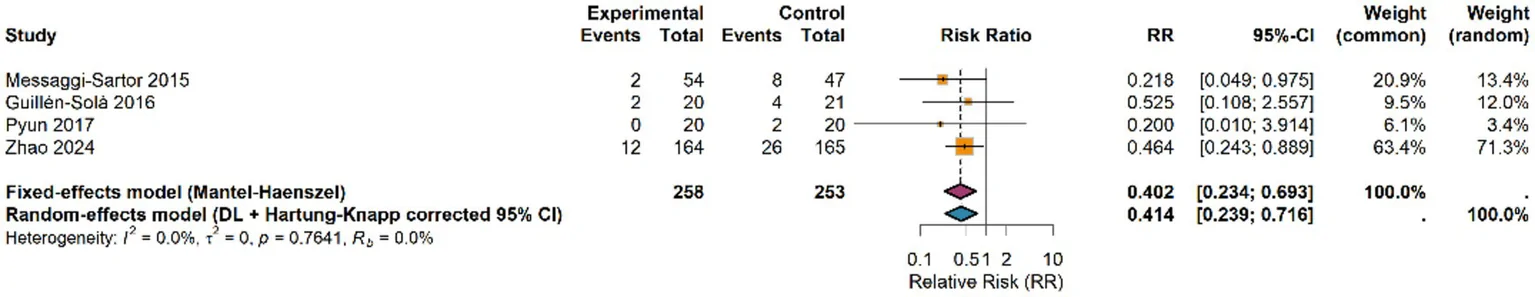
Forest plot of pneumonia incidence.

### Trunk impairment scale

Three RCTs (*n* = 447) provided Trunk Impairment Scale (TIS) data. Higher TIS scores indicate better trunk control. The intervention was associated with a significant improvement in TIS scores (pooled MD = 2.274, 95% CI 1.122–3.426, *p* = 0.0001, Table 2; Figure 8). Between-study heterogeneity was moderate (I^2^ = 56.0%; Q-test *p* = 0.1032). In leave-one-out sensitivity analysis, statistical significance was lost after omitting the largest trial, Zhao 2024 (MD = 2.109, 95% CI − 0.047 to 4.265, *p* = 0.0552), suggesting that the overall finding was partly driven by this study. Publication bias was not assessable given the limited number of studies.

**Figure 8:**
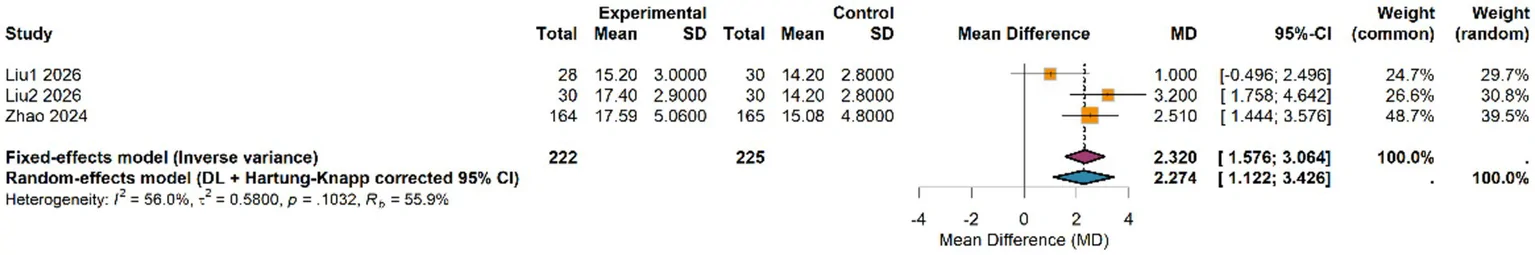
Forest plot of trunk impairment scale.

### Sensitivity analyses

Leave-one-out sensitivity analysis was performed for all meta-analytic outcomes. The plots are provided in the Supplementary materials.

### Publication bias

Publication bias was assessed using funnel plots and Egger’s regression test, with the trim-and-fill method applied as a supplementary analysis. Because Egger’s test is adequately powered and its results are reliable only when at least 10 studies are included, it was performed only for the maximal inspiratory pressure (MIP) outcome, which was the sole outcome comprising ≥10 studies. For outcomes with fewer than 10 studies, Egger’s test *p* values could not be reliably computed; therefore, only funnel plots are presented. Further details are provided in the Supplementary materials.

## Discussion

### Summary of principal findings

This meta-analysis of 11 randomized controlled trials (RCTs) involving 737 subacute stroke patients confirms that respiratory muscle training (RMT) yields significant improvements across respiratory, functional, and clinical endpoints. RMT significantly enhanced inspiratory muscle strength (MIP: MD 14.16 cmH₂O), airway patency (FEV₁: MD 0.32 L), lung volume (FVC: MD 0.31 L), functional exercise capacity (6MWT: MD 34.09 m), and trunk control (TIS: MD 2.27). Critically, RMT reduced pneumonia risk by nearly 60% (RR 0.41). However, the robustness of these pooled estimates varies: FVC (I^2^ = 0%) and pneumonia incidence (I^2^ = 0%) showed striking homogeneity, whereas MIP exhibited substantial heterogeneity (I^2^ = 89.9%), and the effects on 6MWT and TIS were sensitive to single trials. These patterns mandate a nuanced interpretation that moves beyond simple effect-size reporting.

### Comparison with previous meta-analyses and mechanistic insights

#### Consistency and extension of prior evidence

Our estimates for MIP, FEV₁, FVC, and 6MWT align broadly with previous syntheses (26, 31, 32). Notably, Li et al. (17) reported an MIP improvement of 14.68 cmH₂O—nearly identical to our 14.16 cmH₂O—which remarkably reinforces the reproducibility of this finding. We also resolve the previously conflicting evidence on FEV₁: Fabero-Garrido et al. (26) found no significant change, whereas Zhang X et al. (39) and Gomes-Neto et al. (31) reported positive effects. Our meta-analysis demonstrates that FEV₁ significantly improves (MD 0.32 L) specifically in the subacute phase, suggesting that the null result from Fabero-Garrido likely reflects chronic-stage inclusion, where neural plasticity wanes and chest wall compliance declines. This temporal dependency has been overlooked in prior mixed-stage meta-analyses.

#### Pneumonia risk: from uncertainty to robust evidence

Menezes et al. (27) initially reported a significant risk reduction (RR 0.38), but a corrigendum (28) revised this to non-significant (RR 0.49, 95% CI 0.093–2.58). Wu et al. (29) reported a borderline OR 0.55 (*p* = 0.050), vulnerable to small-sample instability. By aggregating 511 patients with 56 events across four trials, we provide the most robust estimate to date (RR 0.41, 95% CI 0.24–0.72) with zero heterogeneity, substantially strengthening the clinical case for RMT in pneumonia prevention. However, it is important to note that Messaggi-Sartor et al. and Guillén-Solà et al. reported composite respiratory outcomes, which cannot be equated with strictly defined pneumonia; Pyun et al. used a relatively strict definition of pneumonia; and Zhao et al. reported pneumonia with significant results but did not clearly define its diagnostic criteria. Therefore, the pooled result should be interpreted as reflecting the risk of “pneumonia, broadly defined (pneumonia or lung infection),” rather than being directly equated with a reduction in the risk of strictly defined pneumonia.

#### The subacute window: a critical temporal niche

Zhang et al. (40) focused on “early stroke” (<3 months) but included acute-phase patients (<2 weeks) who may be medically unstable. Conversely, Huang et al. (34) examined chronic stroke and found no RMT benefit on MEP, FVC, or exercise capacity. Our study occupies the optimal therapeutic window—the subacute phase—where neuroplasticity is most responsive, a distinction that previous syntheses failed to highlight.

#### The FVC–MIP heterogeneity dichotomy: mechanistic implications

A novel insight from our analysis is the stark contrast in heterogeneity between FVC (I^2^ = 0%) and MIP (I^2^ ≈ 90%). Prior meta-analyses often pooled these outcomes in parallel without directly contrasting their heterogeneity profiles. Fabero-Garrido (26) reported high heterogeneity for MIP (I^2^ = 77%) but did not compare it with FVC; Gomes-Neto (31) presented pooled effects without decomposing between-study variance across outcomes; Liu et al. (33) observed substantial heterogeneity for both FVC (I^2^ = 92%) and MIP (I^2^ = 93%) but did not explore this discrepancy. Our empirical observation that FVC is uniformly responsive while MIP is highly variable supports a mechanistic distinction: FVC, reflecting lung volume and chest wall compliance (41), may also be influenced by patient effort and cortical drive (42, 43). The zero heterogeneity indicates that RMT produces a consistent volumetric expansion effect, likely underpinned by structural adaptations—increased diaphragm thickness (MD 0.08 cm) and thickening fraction (MD 47.32%)—as demonstrated by Chen et al. (44), as well as intercostal muscle remodeling (45–47). In contrast, MIP measures maximal static pressure against an occluded airway, which demands maximal voluntary cortical activation and adequate bulbar muscle function (48–50). Its high variability likely reflects differences in training intensity, cognitive status, and measurement timing across studies. Thus, while RMT reliably expands lung volumes, its effect on effort-dependent peak pressures is contingent on patient engagement and protocol specifics—a nuance critical for both trial design and clinical interpretation.

#### Trunk control and respiratory-postural synergy: a novel contribution

Our study is the first to quantitatively synthesize TIS outcomes, showing a mean difference of 2.27, exceeding the minimal clinically important difference (MCID) of 2.09 (51). This finding provides a plausible mechanism linking respiratory and postural functions: the diaphragm and abdominal muscles share fascial continuities with trunk stabilizers via the thoracolumbar fascia (52). RMT likely enhances intra-abdominal pressure regulation, co-activating transversus abdominis and multifidus, thereby improving postural control (53). This is supported by Chen et al. (44), who found RMT improved affected-side diaphragm mobility (MD 1.32 cm), and by correlations between diaphragm function and balance (54). Consequently, the concurrent improvements in 6MWT (+34.09 m) and TIS suggest that RMT facilitates a functional chain from respiratory mechanics to gait performance.

#### Pneumonia reduction: integrating respiratory and swallowing mechanisms

The 59% pneumonia risk reduction likely arises from (a) enhanced cough peak flow (secondary to improved FVC and MIP) facilitating airway clearance, and (b) improved swallowing-respiratory coordination reducing silent aspiration. This aligns with Arnold et al. (55) and Zhang et al. (56), who showed that combined rehabilitation approaches improve swallowing and reduce respiratory complications. Our findings underscore the value of integrating RMT into comprehensive pneumonia prevention strategies for subacute stroke.

### Clinical implications

Clinicians can confidently prescribe RMT to subacute stroke patients to improve pulmonary volumes and lower pneumonia risk. Given the high variability in MIP, we recommend monitoring FVC as a stable and objective marker of training response, while interpreting MIP changes as motivational but effort-dependent. RMT should be combined with task-specific gait and balance training rather than used in isolation. The robust pneumonia reduction suggests that RMT merits inclusion as a core component of infection prevention bundles in subacute stroke units.

### Strengths

This meta-analysis has several methodological strengths. First, it is the first to focus exclusively on the subacute stroke population, a clinically relevant yet under-investigated group. Second, our comprehensive search of six major databases and a trial registry minimized the risk of missing relevant studies. Third, we applied the Hartung-Knapp-corrected random-effects model as the primary analytic approach, which provides more robust estimates with small numbers of studies. Fourth, we performed leave-one-out sensitivity analyses and, where feasible, used trim-and-fill to assess publication bias. Fifth, we systematically evaluated trial quality using the Cochrane Risk of Bias 2 (ROB2) tool and assessed evidence certainty via the GRADE framework, explicitly linking pooled estimates to underlying bias and overall quality—enhancing transparency and interpretability.

### Limitations

Several limitations warrant consideration. First, stroke type and baseline functional status were variably reported across studies; therefore, a pooled description of these characteristics was not feasible, and we could not perform stratified analyses by stroke subtype or baseline severity. This is clinically relevant because prior evidence indicates that stroke type and severity substantially influence both pulmonary function and pneumonia risk. A meta-analysis of 42 studies (26,366 patients) demonstrated that haemorrhagic stroke was associated with a significantly higher risk of post-stroke dysphagia (OR 1.52, 95% CI 1.13–2.07) compared with ischaemic stroke (OR 0.54, 95% CI 0.46–0.65), and dysphagia itself conferred a 4.08-fold increased risk of pneumonia (95% CI 2.13–7.79) (57). Regarding motor impairment, a retrospective study of 134 hemiplegic stroke patients found that FVC declined progressively with worsening trunk control, balance, and activities of daily living; patients with severe trunk impairment (TIS 0–7) had a median FVC of only 63.3%, compared with 94.7% in those with mild impairment (TIS 18–23; *p* = 0.014) (58). Stroke severity, as measured by the NIHSS, has also been consistently identified as an independent predictor of stroke-associated pneumonia. A prospective study of 508 patients reported that NIHSS > 15 was associated with a 9.1-fold increased risk of pneumonia (OR 9.1, 95% CI 5.2–16.0, *p* < 0.01) (59), while a more recent cohort of 847 patients confirmed a dose–response relationship, with each 1-point increase in NIHSS conferring an 8% increase in pneumonia risk (OR 1.08, 95% CI 1.03–1.12, *p* < 0.001) (60). These findings suggest that the pooled effects observed in our meta-analysis may mask important subgroup differences, and that patients with haemorrhagic stroke, severe motor impairment, or higher NIHSS scores may derive differential benefit from RMT. Future trials should stratify randomization or report outcomes by stroke type and baseline severity to enable more precise identification of the patient subgroups most likely to benefit. Second, the number of included trials for certain outcomes was small, limiting statistical power and precluding reliable Egger’s tests or publication bias assessment for outcomes with only three or four studies. Third, substantial heterogeneity persisted for MIP and, to a lesser degree, FEV₁, likely due to variations in training protocols, measurement techniques, and participant characteristics; although sensitivity analyses indicated no single trial drove this heterogeneity, pooled MIP estimates should be interpreted with caution. Fourth, the results for 6MWT and TIS were not robust to leave-one-out analysis—significance disappeared after removing one influential study—suggesting these estimates are less reliable. Fifth, intervention characteristics varied widely across trials—including training modality, intensity, frequency, progression protocol, duration, and control conditions—thereby contributing to heterogeneity. For example, some trials used combined inspiratory and expiratory training, whereas others trained the inspiratory muscles alone; training intensity ranged from 30 to 60% MIP; and treatment duration ranged from 3 to 8 weeks. These differences may have influenced the pooled effects on respiratory muscle strength, pulmonary function, and pneumonia/lung infection outcomes. We have acknowledged this heterogeneity as a limitation. It may partially explain the heterogeneity of the pooled results and limits inferences regarding the optimal training protocol. Future studies should therefore adopt standardized intervention protocols to facilitate direct comparisons. Sixth, the limited number of studies precluded subgroup analyses or meta-regression to explore sources of heterogeneity. Seventh, ROB2 assessment revealed methodological limitations, especially in blinding of participants and personnel, and GRADE ratings ranged from low to moderate, reflecting concerns about heterogeneity, imprecision, and risk of bias. Eighth, although the pooled results suggest that respiratory muscle training may reduce the risk of pneumonia in patients with subacute stroke, this finding should be interpreted with caution, particularly regarding its generalizability. The four trials contributing to the pneumonia analysis predominantly enrolled patients within the first 30–60 days post-stroke: Messaggi-Sartor et al. recruited patients within 3 weeks of onset; Guillén-Solà et al. within 1–3 weeks; Yoo et al. within a median of 24 days; and Zhao et al. within 4 weeks. The median time from stroke onset to enrolment across these trials was therefore substantially shorter than the upper boundary of our pre-specified subacute window (6 months). We therefore emphasize that our pneumonia findings should be regarded as most applicable to patients in the early subacute phase (within approximately 2 months post-onset), and that extrapolation to patients in the late subacute or chronic stages should be made with caution until adequately powered trials specifically enrolling these populations become available. In addition, the effect estimate is based on only a small number of RCTs and is accompanied by some degree of clinical and methodological heterogeneity across studies. Further high-quality, large-sample, multicenter randomized controlled trials are therefore needed to verify the robustness of these findings. It is also important to emphasize that the primary outcome of this meta-analysis was pneumonia, broadly defined (pneumonia or lung infection). Although this approach was intended to address heterogeneity in outcome definitions across studies, several limitations warrant consideration: the broadly defined outcome may conflate events of differing clinical severity; some studies did not provide explicit diagnostic criteria for pneumonia; and individual studies generally lacked sufficient power to assess the incidence of pneumonia. Therefore, the pooled results should be interpreted with caution, and future studies should adopt standardized diagnostic criteria for pneumonia. Finally, our findings are specific to subacute stroke and should not be generalized to acute or chronic phases without further evidence.

### Future directions

We call for: (I) large-scale, multi-center RCTs specifically targeting the subacute phase with standardized RMT protocols; (II) direct comparisons of different RMT modalities to identify optimal regimens; (III) inclusion of cough peak flow and swallowing assessments to disentangle pneumonia reduction mechanisms; (IV) individual participant data meta-analyses to test moderators; (V) long-term follow-up (≥6 months) to evaluate sustainability; and (VI) sex-stratified analyses, given emerging evidence of sex differences in respiratory physiology.

## Conclusion

RMT is an evidence-based adjunctive therapy for subacute stroke patients, demonstrating significant improvements in respiratory muscle strength, pulmonary function, functional exercise capacity, trunk control, and pneumonia risk reduction. This is the first meta-analysis to focus exclusively on the subacute phase, demonstrate the FVC–MIP heterogeneity dichotomy, provide robust pneumonia evidence, and quantitatively synthesize TIS outcomes. These novel contributions distinguish our study from all previous syntheses and provide clinicians with a clear, evidence-based rationale for incorporating RMT into subacute stroke rehabilitation protocols. Nevertheless, the current evidence rests on variable-quality trials with substantial heterogeneity for effort-dependent outcomes; caution is warranted until larger confirmatory studies emerge.

## Data Availability

The original contributions presented in the study are included in the article/Supplementary material, further inquiries can be directed to the corresponding author.
