# Supplementary material for: Effects of respiratory muscle training on respiratory function, functional outcomes, and pulmonary complications in patients with stroke: a systematic review and meta-analysis of randomized controlled trials
Source: Front Neurol. 2026 Oct 23;17:1981476. doi: 10.3389/fneur.2026.1981476 (PMC13739504; doi:10.3389/fneur.2026.1981476)
Supplement: Supplementary file 1 [file Supplementary_file_1.docx]

**Methods: Search strategy**

**eTable 1. The GRADE Rating of Maximum Inspiratory Pressure**

**eTable 2. The GRADE Rating of Forced Expiratory Volume in One Second**

**eTable 3. The GRADE Rating of Forced Vital Capacity**

**eTable 4. The GRADE Rating of Six-Minute Walk Test**

**eTable 5. The GRADE Rating of Pneumonia Incidence**

**eTable 6. The GRADE Rating of Trunk Impairment Scale**

**eTable 7. The study-level definitions of subacute stroke for all included trials**

**eTable 8. The detailed baseline data of all trials**

**eFigure 1. Forest plot of Maximum Inspiratory Pressure**

**eFigure 2. Forest plot of Forced Expiratory Volume in One Second**

**eFigure 3. Forest plot of Forced Vital Capacity**

**eFigure 4. Forest plot of Six-Minute Walk Test**

**eFigure 5. Forest plot of Pneumonia Incidence**

**eFigure 6. Forest plot of Trunk Impairment Scale**

**eFigure 7. Sensitivity analysis of Maximum Inspiratory Pressure**

**eFigure 8. Sensitivity analysis of Forced Expiratory Volume in One Second**

**eFigure 9. Sensitivity analysis of Forced Vital Capacity**

**eFigure 10. Sensitivity analysis of Six-Minute Walk Test**

**eFigure 11. Sensitivity analysis of Pneumonia Incidence**

**eFigure 12. Sensitivity analysis of Trunk Impairment Scale**

**eFigure 13. Funnel plot of Maximum Inspiratory Pressure**

**eFigure 14. Funnel plot of Forced Expiratory Volume in One Second**

**eFigure 15. Funnel plot of Forced Vital Capacity**

**eFigure 16. Funnel plot of Six-Minute Walk Test**

**eFigure 17. Funnel plot of Pneumonia Incidence**

**eFigure 18. Funnel plot of Trunk Impairment Scale**

**Methods: Search strategy**

ClnicalTrials.gov:

Search Details: Viewing 1-10 out of 330 studies for: Stroke OR Cerebrovascular OR 'Vascular Accident' OR Apoplexy OR CVA OR 'Cerebral hemorrhage' OR 'Cerebral infarction' OR Hemiplegia OR Monoplegia | 'Respiratory muscle training' OR Respirator OR Inspiratory OR Expiratory

**Pubmed:**

| No. | Query | Results |
| --- | --- | --- |
| #1 | (((((((((Stroke[MeSH Terms]) OR (Stroke[Title/Abstract])) OR (Cerebrovascular[Title/Abstract])) OR (Vascular Accident[Title/Abstract])) OR (Apoplexy[Title/Abstract])) OR (CVA[Title/Abstract])) OR (Cerebral hemorrhage[Title/Abstract])) OR (Cerebral infarction[Title/Abstract])) OR (Hemiplegia[Title/Abstract])) OR (Monoplegia[Title/Abstract]) | 511,234 |
| #2 | ((((Respiratory muscle training[MeSH Terms]) OR (Respiratory muscle training[Title/Abstract])) OR (Respirator[Title/Abstract])) OR (Inspiratory[Title/Abstract])) OR (Expiratory[Title/Abstract]) | 84752 |
| #3 | Trial[Title/Abstract] | 985,559 |
| #4 | #1 AND #2 AND #3 | 176 |
| #5 |  |  |
| #6 |  |  |

**Embase:**

| No. | Query | Results |
| --- | --- | --- |
| #1 | Stroke:ab,ti,kw OR Cerebrovascular:ab,ti,kw OR 'Vascular Accident':ab,ti,kw OR Apoplexy:ab,ti,kw OR CVA:ab,ti,kw OR 'Cerebral hemorrhage':ab,ti,kw OR 'Cerebral infarction':ab,ti,kw OR Hemiplegia:ab,ti,kw OR Monoplegia:ab,ti,kw | 771278 |
| #2 | 'Respiratory muscle training':ab,ti,kw OR Respirator:ab,ti,kw OR Inspiratory:ab,ti,kw OR Expiratory:ab,ti,kw | 119404 |
| #3 | Trial:ab,ti,kw | 1666971 |
| #4 | #1 AND #2 AND #3 | 299 |
| #5 |  |  |
| #6 |  |  |
|  |  |  |

**Web of Science:**

| No. | Query | Results |
| --- | --- | --- |
| #1 | TS=(Stroke OR Cerebrovascular OR 'Vascular Accident' OR Apoplexy OR CVA OR 'Cerebral hemorrhage' OR 'Cerebral infarction' OR Hemiplegia OR Monoplegia) | 667,787 |
| #2 | TS=('Respiratory muscle training' OR Respirator OR Inspiratory OR Expiratory) | 83,981 |
| #3 | TS=Trial | 2,391,944 |
| #4 | #1 AND #2 AND #3 | 312 |
| #5 |  |  |
| #6 |  |  |
|  |  |  |

**Cochrane library**

| No. | Query | Results |
| --- | --- | --- |
| #1 | (Stroke OR Cerebrovascular OR 'Vascular Accident' OR Apoplexy OR CVA OR 'Cerebral hemorrhage' OR 'Cerebral infarction' OR Hemiplegia OR Monoplegia):ab,ti,kw | 100085 |
| #2 | ('Respiratory muscle training' OR Respirator OR Inspiratory OR Expiratory):ab,ti,kw | 35460 |
| #3 | Trial:ab,ti,kw | 1236715 |
| #4 | #1 AND #2 AND #3 | 522 |
| #5 |  |  |
| #6 |  |  |

**Scopus**

| No. | Query | Results |
| --- | --- | --- |
| #1 | TITLE-ABS-KEY(Stroke OR Cerebrovascular OR 'Vascular Accident' OR Apoplexy OR CVA OR 'Cerebral hemorrhage' OR 'Cerebral infarction' OR Hemiplegia OR Monoplegia) | 73450 |
| #2 | TITLE-ABS-KEY('Respiratory muscle training' OR Respirator OR Inspiratory OR Expiratory) | 20741 |
| #3 | TITLE-ABS-KEY(Trial) | 3,428,284 |
| #4 | #1 AND #2 AND #3 | 10 |
| #5 |  |  |
| #6 |  |  |

**eTable 1. The GRADE Rating of Maximum Inspiratory Pressure**

**eTable 2. The GRADE Rating of Forced Expiratory Volume in One Second**

**eTable 3. The GRADE Rating of Forced Vital Capacity**

**eTable 4. The GRADE Rating of Six-Minute Walk Test**

**eTable 5. The GRADE Rating of Pneumonia Incidence**

**eTable 6. The GRADE Rating of Trunk Impairment Scale**

**eTable 7. The study-level definitions of subacute stroke for all included trials**

| Trial | Country | Authors’ phase label | Inclusion time window | Reported stroke duration | Final study-level definition used in this review |
| --- | --- | --- | --- | --- | --- |
| Messaggi-Sartor et al. 2015 | Spain | Subacute | <3 weeks | Mean 9.2 days | Early subacute (<3 weeks) |
| Guillén-Solà et al. 2016 | Spain | Subacute | 1–3 weeks | Mean 10.4 days | Early subacute (1–3 weeks) |
| Sutbeyaz et al. 2010 | Turkey | Subacute | Within previous 12 months | Mean 155–163 days | Subacute-to-chronic; mean ~5 months |
| Pyun et al. 2017 | Korea | Stroke rehabilitation | Within 3 months | Median 24 days (11–95) | Subacute (≤3 months) |
| Bang et al. 2017 | Korea | Subacute | Within 6 months | Mean 3.2 months | Subacute (≤6 months) |
| Zhu et al. 2024 | China | Stroke | Within previous 6 months | Mean ~3 months | Subacute (≤6 months) |
| Defi et al. 2021 | Indonesia | Subacute | 2 weeks–3 months | Not reported | Subacute (14 days–3 months) |
| Liu et al. 2026 | China | Subacute/chronic | 1–12 months | Mean 3.1–3.4 months | Mixed, predominantly subacute; sensitivity analysis performed |
| Akçay et al. 2026 | Turkey | Subacute ischaemic stroke | >1 month | Mean 71–98 days | Subacute (>1 month; mean ~3 months) |
| Zhao et al. 2024 | China | First stroke | ≤4 weeks | Not reported | Early subacute (≤4 weeks) |
| Tovar-Alcaraz et al. 2021 | Spain | Subacute | Within 6 months | Mean 84–89 days | Subacute (≤6 months) |

**eTable 8. The detailed baseline data of all trials**

| Study | Training modality | Device | Intensity | Frequency | Session protocol | Progression | Treatment duration |
| --- | --- | --- | --- | --- | --- | --- | --- |
| Messaggi-Sartor 2015 | Combined (inspiratory + expiratory) | Orygen-Dual Valve | 30% of maximal respiratory pressures; increased weekly by 10 cmH₂O | Twice daily, 5 days/week | 5 sets × 10 breaths, with 1–2 min unloaded recovery breathing between sets | Increased weekly by 10 cmH₂O as tolerated | 3 weeks |
| Guillén-Solà 2016 | Combined (inspiratory + expiratory) | Oxygen Dual Valve | 30% of maximal inspiratory/expiratory pressures; increased weekly by 10 cmH₂O | Twice daily, 5 days/week | 5 sets × 10 breaths, with 1 min unloaded recovery between sets | Increased weekly by 10 cmH₂O | 3 weeks |
| Pyun et al. | Combined (inspiratory + expiratory + breath stacking) | Incentive spirometer (inspiration); Acapella PEP (expiration) | Not reported | Twice daily, 7 days/week | Breath stacking 10 min + inspiratory training 10 min + expiratory training 10 min; total 30 min | Not reported | 3 weeks |
| Sutbeyaz 2010 | Inspiratory muscle training | Threshold IMT | Started at 40% MIP; progressed to 60% MIP | Twice daily, 6 days/week | 15 min per session | Increased by 5–10% per session up to 60% MIP | 6 weeks |
| Bang 2017 | Inspiratory muscle training | Threshold | 30% MIP | Once daily, 5 days/week | 30 min (6 sets × 5 min) | Not reported | 4 weeks |
| Zhu 2024 | Inspiratory muscle training (+ EDP) | POWERbreathe K5 | Initial 30% MIP | Once daily, 6 days/week | 30 inspiratory efforts in 2 sets; 1 min rest between sets | Not reported | 4 weeks |
| Defi 2021 | Inspiratory muscle training | IMT device (unspecified) | 40% MIP | 5 days/week | 5 sets × 15 breaths; 1 min rest between sets; ~15 min | Not reported | 8 weeks |
| Liu 2026 | Inspiratory muscle training | Threshold load device | 50% MIP (sham: 10% MIP) | Three times daily (morning, afternoon, evening), 5 days/week | 5 sets × 10 breaths; 1 min rest between sets; ~15 min | MIP re-measured weekly and adjusted to 50% of new MIP; unstable group progressively increased instability | 4 weeks |
| Akçay 2026 | Inspiratory muscle training | PowerBreathe Classic | Started at 30% MIP; increased weekly by 5 cmH₂O | Twice daily, 5 days/week | 8 breathing cycles, each followed by 4–5 controlled breaths; 15 min total | Increased weekly by 5 cmH₂O as tolerated | 6 weeks (30 sessions) |
| Zhao 2024 | Inspiratory muscle training | Respiratory endurance trainer (Model S2) | 30% MIP; MIP re-measured every 2 weeks | Two sets daily, 5 days/week | 30 breaths per set; 2 sets daily | Adjusted every 2 weeks based on MIP | 4 weeks |
| Tovar-Alcaraz 2021 | Inspiratory muscle training | Threshold IMT / Powerbreathe | Progressive from 15% to 60% MIP | Once daily, 5 days/week | Not reported | Progressive increase to 60% MIP | 8 weeks |

Abbreviations: EDP, external diaphragmatic pacing; IMT, inspiratory muscle training; MIP, maximal inspiratory pressure; PEP, positive expiratory pressure.

Note: “Not reported” indicates that the original article did not provide sufficient information on that intervention parameter.

**eFigure 1. Forest plot of Maximum Inspiratory Pressure**

**eFigure 2. Forest plot of Forced Expiratory Volume in One Second**

**eFigure 3. Forest plot of Forced Vital Capacity**

**eFigure 4. Forest plot of Six-Minute Walk Test**

**eFigure 5. Forest plot of Pneumonia Incidence**

**eFigure 6. Forest plot of Trunk Impairment Scale**

**eFigure 7. Sensitivity analysis of Maximum Inspiratory Pressure**

**eFigure 8. Sensitivity analysis of Forced Expiratory Volume in One Second**

**eFigure 9. Sensitivity analysis of Forced Vital Capacity**

**eFigure 10. Sensitivity analysis of Six-Minute Walk Test**

**eFigure 11. Sensitivity analysis of Pneumonia Incidence**

**eFigure 12. Sensitivity analysis of Trunk Impairment Scale**

**eFigure 13. Funnel plot of Maximum Inspiratory Pressure**

**eFigure 14. Funnel plot of Forced Expiratory Volume in One Second**

**eFigure 15. Funnel plot of Forced Vital Capacity**

**eFigure 16. Funnel plot of Six-Minute Walk Test**

**eFigure 17. Funnel plot of Pneumonia Incidence**

**eFigure 18. Funnel plot of Trunk Impairment Scale**
